# Identification and characterization of novel human tissue-specific RFX transcription factors

**DOI:** 10.1186/1471-2148-8-226

**Published:** 2008-08-01

**Authors:** Syed Aftab, Lucie Semenec, Jeffrey Shih-Chieh Chu, Nansheng Chen

**Affiliations:** 1 Department of Molecular Biology and Biochemistry, Simon Fraser University, 8888 University Drive, Burnaby, BC, V5A 1S6, Canada

## Abstract

**Background:**

Five regulatory factor X (RFX) transcription factors (TFs)–RFX1-5–have been previously characterized in the human genome, which have been demonstrated to be critical for development and are associated with an expanding list of serious human disease conditions including major histocompatibility (MHC) class II deficiency and ciliaophathies.

**Results:**

In this study, we have identified two additional RFX genes–RFX6 and RFX7–in the current human genome sequences. Both RFX6 and RFX7 are demonstrated to be winged-helix TFs and have well conserved RFX DNA binding domains (DBDs), which are also found in winged-helix TFs RFX1-5. Phylogenetic analysis suggests that the RFX family in the human genome has undergone at least three gene duplications in evolution and the seven human RFX genes can be clearly categorized into three subgroups: (1) RFX1-3, (2) RFX4 and RFX6, and (3) RFX5 and RFX7. Our functional genomics analysis suggests that RFX6 and RFX7 have distinct expression profiles. RFX6 is expressed almost exclusively in the pancreatic islets, while RFX7 has high ubiquitous expression in nearly all tissues examined, particularly in various brain tissues.

**Conclusion:**

The identification and further characterization of these two novel RFX genes hold promise for gaining critical insight into development and many disease conditions in mammals, potentially leading to identification of disease genes and biomarkers.

## Background

The regulatory factor X (RFX) gene family transcription factors (TFs) were first detected in mammals as the regulatory factor that binds to a conserved *cis*-regulatory element called the X-box motif about 20 years ago [1]. The X-box motifs, which are typically 14-mer DNA sequences, were initially identified as a result of alignment and inspection of the promoter regions of major histocompatibility complex (MHC) class II genes for conserved DNA elements [2,3]. Further investigations revealed that the X-box motif is highly conserved in the promoter regions of various MHC class II genes [4]. The first RFX gene (RFX1) was later characterized as a candidate major histocompatibility complex (MHC) class II promoter binding protein [5]. RFX1 was later found to function also as a transactivator of the hepatitis B virus enhancer [6]. Subsequent studies revealed that RFX1 is not alone. Instead, it became the founding member of a novel family of homodimeric and heterodimeric DNA-binding proteins, which also includes RFX2 and RFX3 [7]. More members of this gene family were subsequently identified. A fourth RFX gene (RFX4) was discovered in a human breast tumor tissue [8] and the fifth, RFX5, was identified as a DNA-binding regulatory factor that is mutated in primary MHC class II deficiency (bare lymphocyte syndrome, BLS) [9]. The identification of RFX1-5 and RFX genes in other genomes including the genomes of lower eukaryote species *Saccharomyces cerevisiae *[10] and *Schizosaccharomyces pombe *[11], and higher eukaryote species the nematode *Caenorhabdits elegans *[12] helped understand both the evolution of the RFX gene family and the DNA binding domains [13]. Notably, while previous studies reported five RFX genes (RFX1-5) in human, only one RFX gene has been identified in most invertebrate animals and yeast. In contrast, the fruit fly (*Drosophila melanogaster*) genome has been found to have two RFX genes, dRFX [14] and dRFX2 [15]. All of these RFX genes are transcription factors possessing a novel and highly conserved DNA binding domain (DBD) called RFX DNA binding domain [13], the defining feature of all members belonging to the RFX gene family, suggesting that these RFX TFs all bind to the X-box motifs.

In addition to the defining DBD domains in all of these RFX genes, most of these previously identified RFX genes also contain other conserved domains including B, C, and D domains [13]. The D domain is also called the dimerization domain [13]. The B and C domains also play a role in dimerization and are thus called the extended dimerization domains [16]. Another important domain found in many members of the RFX family is the RFX activation domain (AD). For instance, RFX1 contains a well defined AD [16]. However, AD is not found in many other members of the RFX family including the human RFX5 and *C. elegans *DAF-19 [13]. Outside of these conserved domains, RFX genes from different species or even from same species show little similarity in other regions, which is quite consistent with their diverse functions and distinct expression profiles.

In humans, RFX1 is primarily found in the brain with high expression in cerebral cortex and Purkinje cells [17]. RFX2 [18] and RFX4 [19] are found to be heavily expressed in the testis. RFX4 is also expressed in the brain [20]. RFX3 is expressed in ciliated cells and is required for growth and function of cilia including pancreatic endocrine cells [21], ependymal cells [22], and neuronal cells [23]. RFX3-deficient mice show left-right (L-R) asymmetry defects [23], developmental defect, diabetes [21], and congenital hydrocephalus in mice [22]. RFX5 is the most extensively studied RFX gene so far primarily since it serves as a transcription activator of the clinically important MHC II genes [24] and mediates a enhanceosome formation, which results in a complex containing RFXANK (also known as RFX-B), RFXAP, CREB, and CIITA [25]. Mutation in any one of these complex members leads to bare lymphocyte syndrome (BLS) [25]. In *C.elegans *and *S.cerevisae *only one copy of the RFX gene exists. In *C. elegans *it is called DAF-19 and in *S.cerevisae *it is called Crt1. DAF-19 is involved in regulation of sensory neuron cilium whereas Crt-1 is involved in regulating DNA replication and damage checkpoint pathways [10,12]. In *D.melanogaster*, two of RFX genes have been identified, one is called dRFX and the other is called dRFX2. dRFX is expressed in the spermatid and brain and is necessary for ciliated sensory neuron differentiation [14,26]. dRFX2 has not been studied extensively and as such its function in Drosophila still remains unclear; however, there is evidence suggesting that dRFX2 plays a role in cell-cycle of the eye imaginal discs [15].

In this project, we have identified and characterized two novel RFX genes in genomes of human and many other mammals, which have now been sequenced, annotated, and analyzed.

## Results and discussions

With the current version of the human genome [27,28], we explored whether additional members of the RFX TF family could be identified and characterized in the human genome. We applied a Hidden Markov Model (HMM) based search method [29] and used DBD domain sequences of known human RFX TFs to search the entire human proteome. In addition to retrieving all known human RFX genes–RFX1-5, we identified two additional genes in the human genome that contain well conserved RFX DBDs. These two genes were previously assigned as RFXDC1 and RFXDC2 by the HUGO Gene Nomenclature Committee (HGNC, http://www.genenames.org/); this nomenclature was based solely on an initial bioinformatic analyses. There are no previous publications describing these two genes. Here, we demonstrate that these two genes are also RFX gene family members closely related to RFX1-5, and our phylogenetic analysis suggests two separate recent gene duplications leading to the generation of these two genes. Thus, we proposed new gene nomenclature of RFX6 and RFX7 (Table 1), respectively. Our proposal has been accepted by the HGNC.

**Table 1:** Names and Protein ID of Representative RFX genes.

Gene names	Accession Number (RefSeq)	ESEMBL protein ID	Genomic coordinates				Protein lengths	Number of exons	Number of isoforms
						
			chromosome	start	end	strand			
RFX1	NM_002918	ENSP00000254325	19	13933353	13978097	-1	979	21	1
RFX2	NM_000635	ENSP00000306335	19	5944175	6061554	-1	723	18	2
RFX3	NM_134428	ENSP00000371434	9	3208297	3515983	-1	749	18	8
RFX4	NM_213594	ENSP00000350552	12	105501163	105680710	1	744	18	4
RFX5	NM_000449	ENSP00000357864	1	149581060	149586457	-1	616	11	3
RFX6	NM_173560	ENSP00000332208	6	117305068	117351384	1	928	19	2
RFX7	NM_022841	ENSP00000373793	15	54166958	54222377	-1	1281	7	1

Because all known human RFX genes–RFX1-5–are well conserved and have been identified in other mammalian genomes, we hypothesized that orthologs of RFX6 and RFX7 also exist in other mammalian genomes. As expected, we have retrieved all seven RFX genes in the genomes of five other mammalian species including chimpanzee (*Pan troglodytes*), monkey (*Macaca mulatta*), dog (*Canis familiaris*), mouse (*Mus musculus*), and rat (*Rattus norvegicus*) with only one exception. In the rat genome, all except RFX2 were found despite extensive searches (Additional file 1). Most identified RFX genes are expressed and their transcripts can be found in existing EST libraries. Interestingly, existing EST evidence suggests that RFX6 and RFX7 have no or very few alternative isoforms similar to RFX1. In contrast, RFX2-4 usually have more alternative isoforms (Additional file 1).

**Figure 1:**
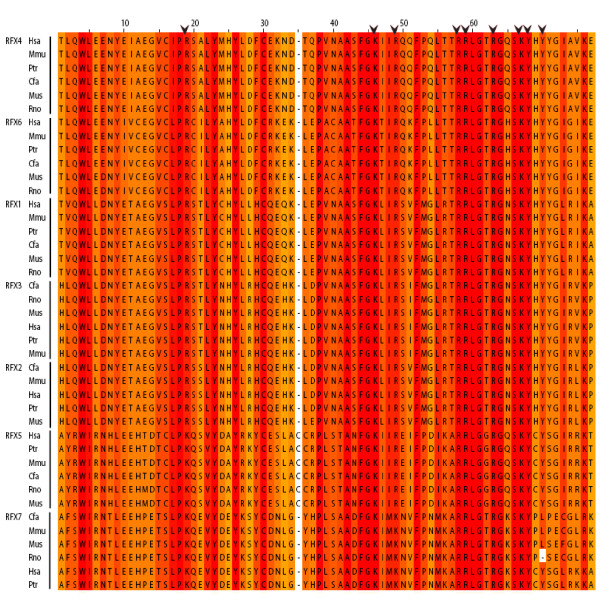
**Mammalian RFX DBDs are highly conserved**. DBDs from six mammalian RFX genes were aligned using ClustalW. The conservation of amino acid is depicted by a color gradient from the color yellow, which indicates low conservation, to red, which indicates high conservation. Nine residues that make direct or water-mediated DNA contacts are indicated with arrow heads. The species names included in this figure are abbreviated. They are: Mus–mouse (*Mus musculus*); Rno–Rat (*Rattus norvegicus*); Cfa–dog (*Canis familiaris*); Ptr–chimpanzee (*Pan troglodytes*); Mmu–monkey (*Macaca mulatta*) and Hsa–human (*Homo sapiens*).

To confirm that the two novel human RFX genes–RFX6 and RFX7 are indeed RFX TFs, we further examined their DBDs by aligning them with DBDs from RFX1-5 protein sequences. As expected, the DBDs of RFX6 and RFX7 align well with those of RFX1-5 (Figure 1). RFX TFs belong to the winged-helix family of DNA binding proteins because their DBDs are related in structure and function to the helix-turn-helix bacterial transcriptional regulatory proteins [30]. DBDs from RFX6 and RFX7 each contain one wing (W1), which is the same as DBDs from RFX1-5. W1 interacts with the major groove and another conserved fold H3 (helix 3) interacts with the minor groove of DNA. In particular, the nine residues in DBDs (Figure 1, indicated with arrow heads) that make direct or water-mediated DNA contacts [31] are almost entirely conserved in RFX6 and RFX7 (Figure 1) with a couple of minor exceptions. Of the nine residues, the human RFX7 DBD has two residues different from most of the other RFX DBDs. The first different residue is the first of the nine indicated residues. It is Lys in RFX7 DBD and RFX5 DBD, compared to Arg in DBDs of other RFX genes. Thus this difference is shared with the RFX5 DBD. The other different residue is the third of the nine residues. It is Lys in RFX7, compared to Arg at this site for DBDs of all other RFX genes. Because both Lys and Arg are basic amino acids, such substitutions are not expected to have dramatic impacts on the binding between the DBDs and their cognate binding sites. This high degree of conservation suggests that RFX6 and RFX7 may bind to similar if not identical *cis*-regulatory elements, i.e., the X-box motif [1]. Hence RFX6 and RFX7 are new members of the human RFX gene family with conserved DBDs.

**Figure 2:**
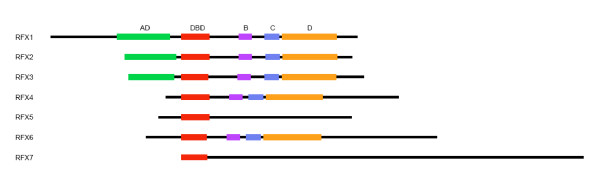
**Functional domains in the known and novel human RFX genes**. The functional domains, AD, DBD, B, C, and D are indicated using color-coded boxes. Genes are represented using horizontal lines, which are proportional to the protein lengths. The domain lengths and positions are also proportional to their actual lengths. The graphs are aligned based on the position of the DBDs.

In addition to the highly conserved DBDs, other domains including ADs, B, C, and D domains (also known as dimerization domain) [13] have been described in human RFX1-3 (Figure 2). Among these functional domains, ADs have been identified in RFX1-3. However, ADs have not been identified RFX4-5. The B and C domains, which are usually called extended dimerization domains, play supporting roles in dimerization [16]. B, C, and D domains have also been identified in RFX4 but are missing from RFX5. Using InterProScan [32] and HMMER [29], we have found that RFX6 possesses B, C, and D domains, but not AD (Figure 2). The motif composition of RFX6 is similar to RFX4, which also has B, C, and D domains but lacks AD. In contrast, we failed to identify B, C, and D domains or AD in RFX7. None of these domains can be found in RFX5 as well. Because these C-terminal domains–B, C, and D domains–have been shown to mediate dimerization as well as transcriptional repression [33], RFX6, which contains B, C, D domain, and RFX7, which does not possess B, C, or D domains, may therefore play different role in transcriptional regulation.

**Figure 3:**
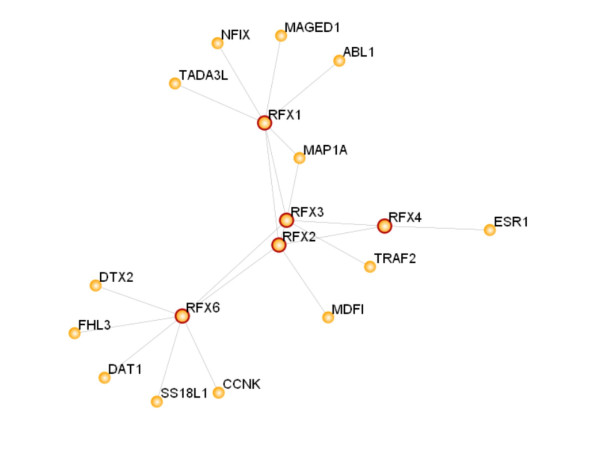
**RFX interactome**. Circles depict gene products and lines depict protein-protein interactions. The interactions between RFX6 and its direct interactors were obtained using yeast-two-hybrid method in a large-scale human protein-protein interaction study [34]. Additional interactions were constructed by Rhodes *et al*[46]. The network was generated using program available at the HiMap website http://www.himap.org/[46].

Characterization of the functional domain composition of RFX genes will provide insights into how different RFX TFs function. In particular, how do RFX6 and RFX7, as well as RFX4 and RFX5, function in transcription considering that they do not have identified ADs? There are two possible mechanisms. First, because RFX TFs are known to form dimers and bind to same or similar binding sites (the X-box motifs) in DNA [31], they may function together with RFX genes (RFX1-3) that do have ADs. Examination of a recently available proteome-scale map of the human protein-protein interaction network [34], which was constructed using yeast-two-hybrid technique, has shown that RFX6 and RFX1-4 interact with each other and also interact with many other genes (Figure 3). RFX6 interacts directly with RFX2 and RFX3, the latter of which has been shown to be expressed and to function in the pancreas [21], as well as many other tissues. The interaction between RFX6 and other RFX TFs provides further supporting evidence that RFX6 is indeed a member of the RFX gene family. Interactions between RFX7 and other genes were not observed, which is likely due to the incomplete coverage of the human protein-protein interactions analyzed in this study. Second, RFX TFs may function by interacting with many other non-RFX TFs. For example, it has been demonstrated that mammalian RFX 5 forms a complex ("enhanceosome") with RFXANK (also known as RFX-B), RFXAP, CREB, and CIITA to regulate expression of MHC class II genes [25]. Notably, all of the five genes shown to interact with RFX6 (DTX1, DTX2, FHL3, CCNK, and SS18L1) (Figure 3) except only one–SS18L1–are also putative TFs.

**Figure 4:**
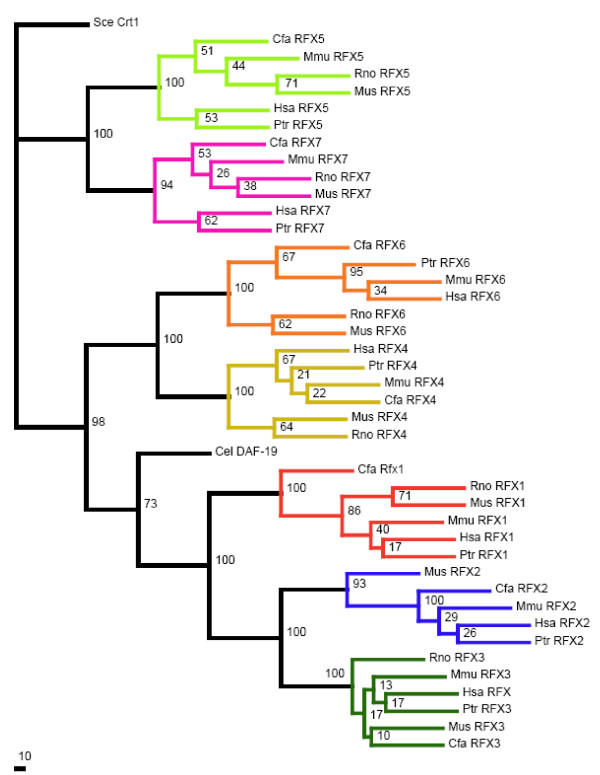
**Phylogenetic analysis of mammalian RFX genes**. This phylogenetic tree was constructed based on DBDs of RFX genes for six mammalian species and *C. elegans *using yeast RFX gene product Crt1 as the out-group. The phylogenetic tree was bootstrapped for 100 times with the numbers at each internal node being the bootstrap values. Each ortholog group is colored differently. The species names included in this figure are abbreviated. They are: Mus–mouse (*Mus musculus*); Rno–Rat (*Rattus norvegicus*); Cfa–dog (*Canis familiaris*); Ptr–chimpanzee (*Pan troglodytes*); Mmu–monkey (*Macaca mulatta*) and Hsa–human (*Homo sapiens*).

To explore the relationship between RFX6 and RFX7 and the known RFX family members RFX1-5, we have constructed a phylogenetic tree that contains all mammalian RFX genes described above (Additional file 1, Figure 1), as well as *C. elegans *RFX gene *daf-19 *product DAF-19 [12], which has been extensively studied, for comparison. We used the DBD sequence of the yeast *Saccharomyces cerevisiae *RFX gene Crt-1[10] as an out group in the phylogenetic tree construction. From the phylogenetic tree (Figure 4), all seven genes show perfect one-to-one orthologous relationships between different mammalian genomes. It is clear that the seven mammalian RFX genes fall into three subgroups (Figure 4). The first subgroup contains RFX1-3; the second RFX4 and RFX6; while the third RFX5 and RFX7. It is likely that RFX4 and RFX6 resulted from one gene duplication that predated the split of these mammalian species, while RFX5 and RFX7 resulted from another similar independent duplication. This hypothesis is generally consistent with the gene models of these RFX genes (Additional file 2). RFX6 has 19 exons, which is similar to the number of exons contained in RFX4 (18 exons); while RFX7 has 6 exons, which is similar to the number of exons contained in RFX5 (9 exons). The *C. elegans *RFX gene, DAF-19 clusters together with RFX1-3 genes, supporting a previously proposed hypothesis that the divergence of the subgroup RFX1-3 from other two subgroups likely predated the divergence between mammals and the nematodes [13]. This hypothesis predicts that *C. elegans *should have orthologous RFX TFs to RFX4-7 [35]. However, only one *C. elegans *RFX gene–*daf-19*–has been reported so far and our extensive search has concluded that *daf-19 *is the only RFX TF in *C. elegans*. One possible explanation is that additional RFX TFs were lost in evolution. Alternatively, RFX4-7 may have undergone positive selection in mammals to accommodate additional functional complexity in mammalian gene regulation, while RFX1-3 and *daf-19 *remained highly conserved due to purifying evolution. Interestingly, although the phylogenetic tree was constructed based only on DBDs, the grouping of these mammalian RFX genes is also consistent with the composition of other conserved domains. In particular, RFX1-3 all contain DBDs, ADs, Bs, Cs and Ds, while RFX4 and RFX6 have all of these domains except ADs, and RFX5 and RFX7 have only DBDs (Figures 2 and 4).

To gain insight into the function of these two newly identified RFX genes, we explored the expression profiles of RFX6 and RFX7 and compared them to those of RFX1-5. We analyzed two independent datasets. First, we searched the dbEST database in genBank http://www.ncbi.nlm.nih.gov/dbEST/[36] to examine which EST libraries express transcripts of these RFX genes. The results indicate that the expression profile of RFX1-5 matches well with previously published data (see INTRODUCTION): RFX1 is found in many different tissue types including white blood cells, heart, eye, testis, and cancerous cell; RFX2 appears to be expressed in testis and brain; RFX3 appears to be expressed in the placenta and brain (*i.e*., medulla); RFX4 is found in the brain, as well as in testis as RFX2; and RFX5 expression has been observed in various different tissues including thymus, T-cells, kidney, brain, and lymph. The consistency of expression for RFX1-5 obtained from the dbEST database with previous observations suggests that dbEST provides good estimations of RFX genes' expression profiles. Using the same method, we found that RFX6 is primarily expressed in pancreas, with minor expression in liver, while RFX7 is widely and heavily expressed in many different tissue types including kidney (tumor tissues), thymus, brain, and placenta.

**Figure 5:**
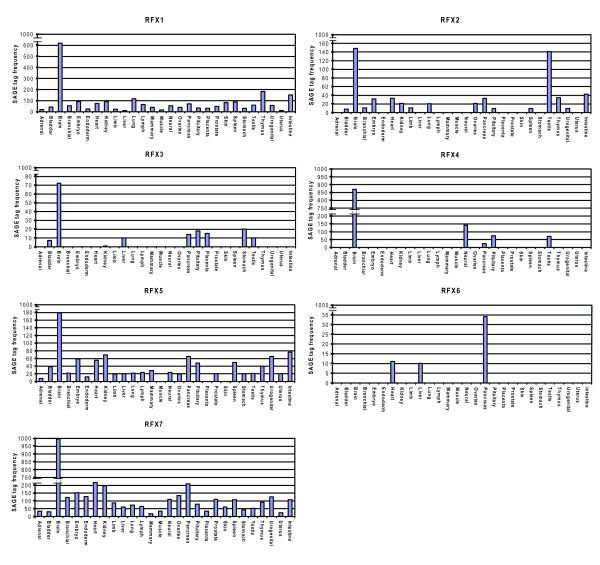
**Relative expression of human RFX genes revealed by SAGE**. Original SAGE libraries were generated by the Mouse Atlas Project [37]. X-axis shows different tissue types, while Y-axis shows relative SAGE tag frequency.

Second, to gain a quantitative understanding of the expression of RFX genes, we took advantage of the recent availability of serial analysis of gene expression (SAGE) libraries constructed by the Mouse Atlas of Gene Expression Project http://www.mouseatlas.org/[37]. To start with, we tested the hypothesis that the expression of mouse RFX TFs approximates the expression of human RFX TFs. We analyzed 196 mouse SAGE libraries, each of which was produced by using a RNA library prepared from different tissue types (some of which are duplicates). Different SAGE libraries contain slightly different number of total SAGE tags. To ensure that SAGE tags and tag counts were comparable between different SAGE libraries all the libraries were normalized to 1,000,000 SAGE tags. Qualitatively, expression profiles of mouse RFX genes obtained from SAGE analysis are consistent with the expression profiles of human RFX genes obtained from the dbEST database analysis, as well as previous publications about human RFX gene expressions (Figure 5). In contrast to all other RFX genes–RFX1-5 and RFX7, which are heavily expressed in the brain, RFX6 is clearly absent from all types of brain tissues (Figure 5). RFX6 is primarily found in the pancreas (Figure 5) which is consistent with results obtained from analyzing dbEST. Low level expression of RFX6 is found in liver (also detected in dbEST) and heart. In addition to the high tissue-specificity, RFX6 has the lowest overall expression level among all seven RFX genes, suggesting that RFX6 may be under tighter regulatory control. In contrast, RFX7 has the highest relative expression level among all seven mouse RFX genes. Similar to RFX1 and RFX5, RFX7 is found in essentially all types of tissues that were examined (Figure 5).

Examining additional gene expression databases, including publicly available Genomics Institute of the Novartis Research Foundation (GNF) Gene Expression Database http://symatlas.gnf.org/SymAtlas/, revealed very similar results.

## Conclusion

Our results show that we have identified two novel RFX genes in the human genome, RFX6 and RFX7, thus expanding the human RFX gene family from five members (RFX1-5) to seven members (RFX1-7). In addition to their possession of highly conserved DBDs, RFX6 and RFX7 show similarity to known human RFX TFs in their functional domains. In particular, RFX6 and RFX4 all have B, C, and D domains, while RFX7 and RFX5 only have DBDs. Studies carried out over the past 20 years have demonstrated that RFX1-5 are critical for development and many additional biological processes and play an important role in various devastating disease conditions. For example, RFX3-deficient mice show left-right (L-R) asymmetry defects [23], developmental defects, diabetes [21], and congenital hydrocephalus [22]. RFX3 may regulate the transcription of many genes that, when mutated, cause cilia defects and many disease conditions collectively called ciliopathies [38]. Many known ciliopathy genes, including Bardet-Biedle syndrome (BBS) genes, are well conserved and the transcription of their *C. elegans *orthologs are regulated by the only RFX gene in *C. elegans*–DAF-19 [12,39-41]. Mutation in any one of the RFX5 enhanceosome members–RFXANK, RFXAP, CREB, and CIITA–leads to bare lymphocyte syndrome (BLS) [25]. We hypothesize that RFX6 and RFX7 are equally important as RFX1-5. The fact that RFX6 is primarily expressed in pancreatic tissues and is expressed at a low level compared to all other RFX genes (Figure 5) is particularly interesting. RFX6 may function as a key component of a transcriptional regulatory complex that regulates pancreas development and function.

## Methods

### Data source and data mining

Gene sets were obtained from the FTP site of the ENSEMBL database http://www.ensembl.org/index.html[42]. In this project, the genomes of six mammals were analyzed. They are human (*Homo sapiens*, NCBI36.44), chimpanzee (*Pan troglodytes*, CHIMP2.1.44), dog (*Canis familiaris*, BROADD2.44), monkey (*Macaca mulatta*, MMUL_1.44), mouse (*Mus musculus*, NCBIM36.44), and rat (*Rattus norvegicus*, RGSC3.4.44). DBD sequences in human RFX1-5 were manually identified and extracted to a file. The sequences were aligned using ClustalW [43]. The alignment was used as input to the profile building program hmmbuild, which is a program in the HMMER package http://hmmer.janelia.org[29]. The resulting profile was used for searching curated proteomes of the six mammals described above using hmmsearch, another program in the HMMER package.

### Gene model improvement

All RFX genes except one–dog (Cfa) RFX7–show good alignment with their corresponding orthologs. Dog RFX7 gene is truncated at the N-terminus, missing 37 residues compared to other RFX7 genes. We attempted to use GeneWise http://www.ebi.ac.uk/Wise2/[44,45] to remodel this RFX gene. Using human (Hsa) RFX7 as the reference protein sequence and GeneWise, we recovered the missing residues. However, the first codon so identified was not the typical Met. Extending the coding sequence upstream did not help. This is likely due to a sequencing error.

### Protein domain analysis

We retrieved DBDs and ADs from RFX genes using InterProScan (version 4.3.1) [32]. To identify B, C, D domains, we used the HMMER program [29] as described above. Briefly, for HMMER searches, we used sequences of B, C, and D domains from known RFX genes (RFX1-3) to generate profiles for these domains respectively. We then searched for candidate B, C, and D domains in RFX6 and RFX7 using these profiles.

### RFX interactome network analysis

Data were obtained at the HiMAP http://www.himap.org/ database [46] following online search instructions. All types of interactions were selected for searching. All seven interactions between RFX6 and other genes (DAT1, DTX2, FHL3, SS18L1, CCNK, RFX2, and RFX3) were previously reported by Rual *et al*[34].

### Sequence alignment and phylogenetic analysis

Multiple-sequence alignment was carried out using the program ClustalW (version 1.83) [43]. Phylogenetic tree construction was performed using PHYLIP http://evolution.genetics.washington.edu/phylip.html (Version 3.66). Briefly, sequence alignment in PHYLIP format was first created using ClustalW (Version1.83) [43]. The alignment was used as input for PHYLIP. Programs utilized in the PHYLIP, in their respective order, were seqboot, protdist, neighbor, and consense. The phylogenetic tree file was visualized using Tree View http://taxonomy.zoology.gla.ac.uk/rod/treeview.html.

### Expression profile of mammalian RFX genes using

#### ESTs and SAGE libraries

The EST database from NCBI was used to perform tblastn. The queries used for this tblastn were RFX1-7 of *H. sapiens, M. musculus*, and *R. norvegicus*. Hits with identity greater than or equal to 95% were selected.

## Authors' contributions

NS conceived of the study, participated in experimental design. SA, LS and JSCC carried out the analysis. SA and NS wrote the manuscript. All authors read and approved the final manuscript.

## Supplementary Material

Additional File 1 Gene names and Protein ID of mammalian RFX genes. Click here for file

Additional File 2 Gene models of human RFX genes, including RFX1-5 and newly identified RFX6-7. (a) Exon-intron structures of human RFX genes. Exons are represented using boxes, while introns are represented using lines. Both exons and introns shown in this panel are proportional to their real lengths. (b) Illustration of exon-intron structures of human RFX-genes. In this panel, while exons are proportional to their real lengths, for better visualization, introns are represented using lines of same lengths, regardless of their real lengths. Click here for file
