# Supplementary material for: Identification and characterization of novel human tissue-specific RFX transcription factors
Source: BMC Evol Biol. 2008 Aug 1;8:226. doi: 10.1186/1471-2148-8-226 (PMC2533330; doi:10.1186/1471-2148-8-226)
Supplement: Additional File 1 — Gene names and Protein ID of mammalian RFX genes. [file 1471-2148-8-226-S1.doc]

**Additional table 1:** Gene names and Protein ID of mammalian RFX genes. Data taken from Ensembl (http://www.ensembl.org/)

| **Species** | **Gene Name** | **Accession Number**  **(RefSeq DNA)** | **Protein id** | **Genomic Coordinates** | | | | **Protein  length** | **Number of isoforms** | **Number of exons** |
| --- | --- | --- | --- | --- | --- | --- | --- | --- | --- | --- |
|  |  |  |  | **chr** | **start** | **end** | **strand** |  |  |  |
| Humans  (*Homo sapiens*) | Rfx1 | NM_002918 | ENSP00000254325 | 19 | 13933353 | 13978097 | -1 | 979 | 1 | 21 |
|  | Rfx2 | NM_000635 | ENSP00000306335 | 19 | 5944175 | 6061554 | -1 | 723 | 2 | 18 |
|  | Rfx3 | NM_134428 | ENSP00000371434 | 9 | 3208297 | 3515983 | -1 | 749 | 8 | 18 |
|  | Rfx4 | NM_213594 | ENSP00000350552 | 12 | 105501163 | 105680710 | 1 | 744 | 4 | 18 |
|  | Rfx5 | NM_000449 | ENSP00000357864 | 1 | 149581060 | 149586457 | -1 | 616 | 3 | 11 |
|  | Rfx6 | NM_173560 | ENSP00000332208 | 6 | 117305068 | 117351384 | 1 | 928 | 2 | 19 |
|  | Rfx7 | NM_022841 | ENSP00000373793 | 15 | 54166958 | 54222377 | -1 | 1281 | 1 | 7 |
| Chimpanzee  (*Pan troglodytes*) | Rfx1 | XM_524133 | ENSPTRP00000018049 | 19 | 14361897 | 14409186 | -1 | 979 | 1 | 22 |
|  | Rfx2 | XM_512310 | ENSPTRP00000017613 | 19 | 6101537 | 6159032 | -1 | 699 | 2 | 17 |
|  | Rfx3 | n/a | ENSPTRP00000043155 | 9 | 3260630 | 3528579 | -1 | 715 | 5 | 18 |
|  | Rfx4 | XP_001161826 | ENSPTRP00000040381 | 12 | 107770229 | 107951773 | 1 | 735 | 3 | 18 |
|  | Rfx5 | XM_001171715 | ENSPTRP00000002192 | 1 | 130402119 | 130408780 | -1 | 616 | 1 | 11 |
|  | Rfx6 | XM_527584 | ENSPTRP00000031653 | 6 | 118855615 | 118910695 | 1 | 932 | 1 | 19 |
|  | Rfx7 | XM_001171418 | ENSPTRP00000012142 | 15 | 53515170 | 53604052 | -1 | 1281 | 1 | 9 |
| Dog  (*Canis familiaris*) | Rfx1 | n/a | ENSCAFP00000024289 | 20 | 51497212 | 51519588 | 1 | 954 | 1 | 24 |
|  | Rfx2 | XM_533937 | ENSCAFP00000027631 | 20 | 57014252 | 57054444 | 1 | 727 | 3 | 17 |
|  | Rfx3 | XM_533540 | ENSCAFP00000003031 | 1 | 94845887 | 95002161 | -1 | 755 | 1 | 16 |
|  | Rfx4 | XM_845927 | ENSCAFP00000002647 | 10 | 35119527 | 35257965 | -1 | 745 | 3 | 18 |
|  | Rfx5 | XM_540315 | ENSCAFP00000018636 | 17 | 63473796 | 63477569 | -1 | 619 | 1 | 9 |
|  | Rfx6 | XM_541213 | ENSCAFP00000001307 | 1 | 60291581 | 60346011 | 1 | 923 | 1 | 21 |
|  | Rfx7 | XM_544696 | ENSCAFP00000023611 | 30 | 24303138 | 24347785 | -1 | 1365 | 2 | 6 |
| Monkey  (*Macaca mulatta*) | Rfx1 | n/a | ENSMMUP00000024954 | 19 | 13655404 | 13685077 | -1 | 738 | 1 | 16 |
|  | Rfx2 | n/a | ENSMMUP00000015695 | 19 | 5896888 | 5955543 | -1 | 726 | 2 | 17 |
|  | Rfx3 | n/a | ENSMMUP00000001093 | 15 | 73743838 | 74012735 | 1 | 749 | 6 | 18 |
|  | Rfx4 | n/a | ENSMMUP00000029123 | 11 | 107675355 | 107834734 | 1 | 747 | 2 | 18 |
|  | Rfx5 | n/a | ENSMMUP00000017303 | 1 | 129774114 | 129778573 | -1 | 620 | 1 | 9 |
|  | Rfx6 | n/a | ENSMMUP00000022612 | 4 | 147022168 | 147078490 | -1 | 932 | 1 | 19 |
|  | Rfx7 | n/a | ENSMMUP00000033587 | 7 | 34408030 | 34456302 | -1 | 1364 | 2 | 6 |
| Mouse  (*Mus musculus*) | Rfx1 | NM_009055 | ENSMUSP00000005600 | 8 | 86956971 | 86987107 | 1 | 963 | 1 | 21 |
|  | Rfx2 | n/a | ENSMUSP00000002444 | 17 | 56461024 | 56516132 | -1 | 717 | 2 | 18 |
|  | Rfx3 | NM_011265 | ENSMUSP00000038760 | 19 | 27834136 | 28077137 | -1 | 749 | 3 | 17 |
|  | Rfx4 | NM_001024918 | ENSMUSP00000051107 | 10 | 84185847 | 84336335 | 1 | 735 | 3 | 18 |
|  | Rfx5 | NM_017395 | ENSMUSP00000029772 | 3 | 95039605 | 95046752 | 1 | 658 | 6 | 10 |
|  | Rfx6 | n/a | ENSMUSP00000020054 | 10 | 51366225 | 51418218 | 1 | 931 | 3 | 19 |
|  | Rfx7 | NM_001033536 | ENSMUSP00000091338 | 9 | 72330807 | 72413719 | 1 | 1269 | 1 | 6 |
| Rat  (*Rattus norvegicus*) | Rfx1 | NM_001105944 | ENSRNOP00000008104 | 19 | 25745412 | 25776701 | -1 | 964 | 1 | 21 |
|  | Rfx3 | n/a | ENSRNOP00000019515 | 1 | 231342761 | 231601324 | -1 | 749 | 5 | 17 |
|  | Rfx4 | XM_576205 | ENSRNOP00000056437 | 7 | 20977074 | 21115616 | -1 | 725 | 3 | 17 |
|  | Rfx5 | NM_001107694 | ENSRNOP00000028531 | 2 | 189856029 | 189860951 | 1 | 652 | 1 | 12 |
|  | Rfx6 | NM_001106388 | ENSRNOP00000029472 | 20 | 30335024 | 30390585 | 1 | 931 | 2 | 19 |
|  | Rfx7 | XM_001053787 | ENSRNOP00000008644 | 8 | 77120962 | 77207044 | 1 | 1268 | 2 | 7 |
