# Supplementary figures and images for: Identification and characterization of novel human tissue-specific RFX transcription factors
Source: BMC Evol Biol. 2008 Aug 1;8:226. doi: 10.1186/1471-2148-8-226 (PMC2533330; doi:10.1186/1471-2148-8-226)

a

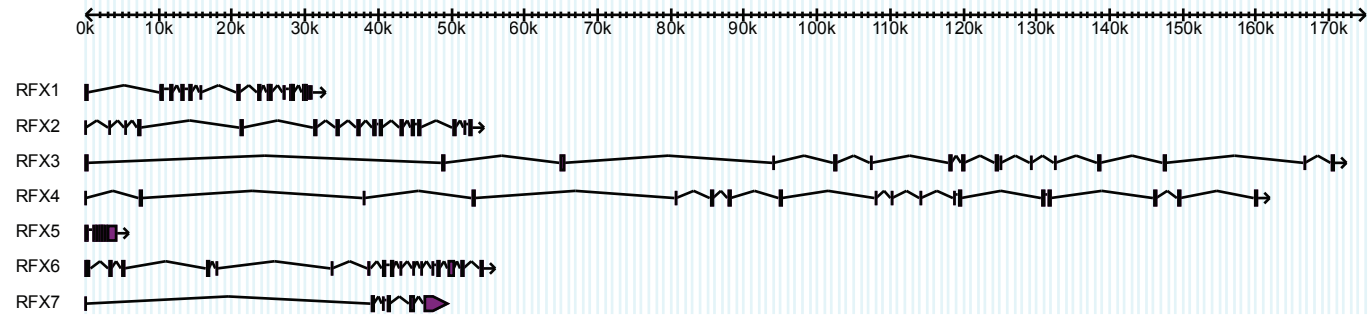

b

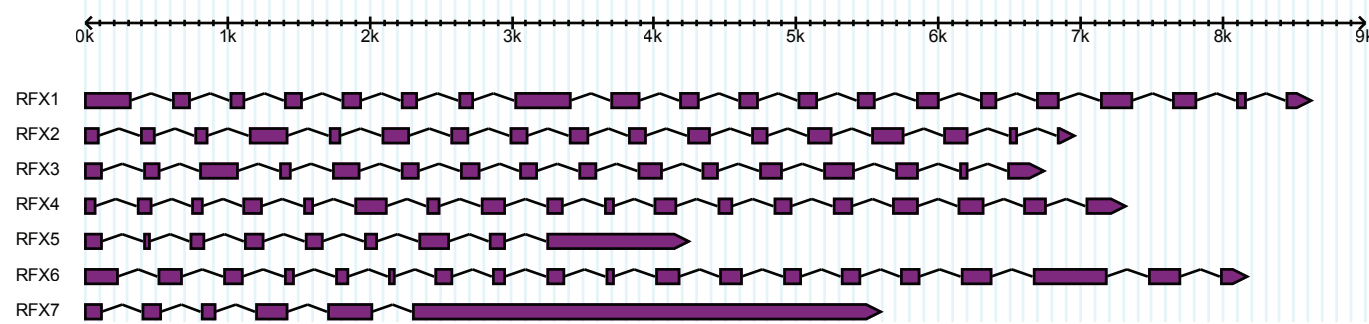

Supplement: Additional File 2 — Gene models of human RFX genes, including RFX1-5 and newly identified RFX6-7. (a) Exon-intron structures of human RFX genes. Exons are represented using boxes, while introns are represented using lines. Both exons and introns shown in this panel are proportional to their real lengths. (b) Illustration of exon-intron structures of human RFX-genes. In this panel, while exons are proportional to their real lengths, for better visualization, introns are represented using lines of same lengths, regardless of their real lengths. [file 1471-2148-8-226-S2.pdf]
